# Siblings with Gorlin–Goltz syndrome associated with cardiac tumors: a case report and review of literature

**DOI:** 10.1186/s13023-023-02792-5

**Published:** 2023-07-05

**Authors:** Paula I. Wilke, Daniel Biermann, Maria Grafmann, Rainer Kozlik-Feldmann, Dzhoy Papingi, Jörg S. Sachweh, Fridrike Stute, Jakob Olfe

**Affiliations:** 1 Pediatric Cardiology, University Medical Center Hamburg-Eppendorf University Heart & Vascular Center, Martinistr. 52, 20246 Hamburg, Germany; 2 Institute of Human Genetics, University Medical Center Hamburg-Eppendorf, Hamburg, Germany; 3 Pediatric Cardiac Surgery, University Medical Center Hamburg-Eppendorf University Heart & Vascular Center, Hamburg, Germany

**Keywords:** Cardiac arrhythmias, Cardiac fibroma, Implantable cardioverter-defibrillator, Gorlin–Goltz syndrome

## Abstract

Primary cardiac tumors in children are very rare and may be associated with severe arrhythmias and sudden infant death syndrome. These cardiac arrhythmias vary depending on the location and size of the tumor. Sixty-four percent of children with cardiac fibroma, the second most common benign cardiac tumor in children, have ventricular arrhythmias, affecting therapeutic management and risk profile of these children. We report on two siblings with cardiac fibromas whose clinical presentations differed depending on their locations and size of the tumors. The first child, a three-year-old girl, was diagnosed with a cardiac fibroma in the left ventricle at the age of 8 months after surviving resuscitation due to ventricular fibrillation. Secondary prophylactic implantation of an ICD was performed. On propranolol, no further malignant arrhythmias have occurred to date. The seven-month-old brother was diagnosed postnatally with a cardiac tumor adjacent to the right ventricle. A few weeks after birth, the boy had refractory supraventricular tachycardia and ventricular arrhythmia that only resolved with amiodarone. In genetic testing, Gorlin–Goltz syndrome was diagnosed in both children. Conservative pharmacological therapy is a therapeutic strategy for asymptomatic patients with cardiac fibromas. The anti-arrhythmic medication depends on the location of the tumor. Implantation of an ICD should be performed in cases of malignant arrhythmias. In rare cases, there is an association between cardiac tumors and genetic syndromes, such as Gorlin–Goltz syndrome. These should always be considered when such a tumor is diagnosed.

**Supplementary Information:**

The online version contains supplementary material available at 10.1186/s13023-023-02792-5.

## Background

Primary cardiac tumors occur very rarely in children with a prevalence between 0.0017 and 0.28%. Ninety percent of the tumors are benign. The most common is rhabdomyoma (60%), followed by fibroma (10–25%), myxoma (6–15%) and teratoma (2–9%). Primary malignant cardiac tumors in childhood are mainly rhabdomyosarcomas and fibromyosarcomas. They occur less frequently than secondary (metastatic) malignant tumors [1].

Clinical symptoms are highly variable depending on the location and size of the tumors. The children may be asymptomatic or present with heart murmurs due to obstruction of the inflow and outflow tracts and ventricular lumen, cyanosis, dyspnea, heart failure, emboli, and arrhythmias. The latter range from single premature beats to ventricular arrhythmias with sudden cardiac death [1]. Larger fibromas are associated with a 50% risk of ventricular tachycardia [2].

Cardiac tumors are usually diagnosed pre- and postnatally by echocardiography. To determine the subtype of the tumor, cardiac MRI or CT is the method of choice. In rare cases, biopsy is indicated if the findings are ambiguous [1].

Depending on the location and size of the tumor and clinical symptoms, antiarrhythmic medication or surgical resection of the tumor should be considered. Inoperable findings may require implantation of an implantable cardioverter-defibrillator or cardiac transplantation [1].

Cardiac fibromas are mostly singular and invasive tumors with a size of 1–9 cm and located intramurally, primarily in the septum and left ventricular apex. Pathognomonic for fibromas are central calcifications and cystic degenerations in the tumor as a consequence of insufficient vascular perfusion [1, 3]. Unlike rhabdomyomas, spontaneous regression of cardiac fibromas is not observed [4].

In rare cases, cardiac tumors are associated with congenital heart defects and genetic syndromes. Rhabdomyomas are seen in 70–90% of cases with tuberous sclerosis. Cardiac fibromas are associated with Beckwith–Wiedemann syndrome and Gorlin–Goltz syndrome [1]. Gorlin–Goltz syndrome is an inherited autosomal dominant disorder often associated with a variant in the protein patched homolog 1 (*PTCH1)* gene on chromosome 9q22. It is associated with the occurrence of basal cell carcinomas, jaw cysts, skeletal abnormalities, calcifications of the falx cerebri, and facial dysmorphia [5]. The occurrence of cardiac fibroma in Gorlin–Goltz syndrome has been described rarely with a frequency of 3–5% [6].

We report on two siblings with cardiac tumors. They developed different, severe cardiac arrhythmias depending on tumor location and size. The first child, a three-year-old girl, was diagnosed with cardiac fibroma in the left ventricle at the age of eight months after surviving resuscitation with observed ventricular fibrillation. Her seven-month-old brother was found to have a cardiac tumor adjacent to the right ventricle after birth. Within the first few months of life, he developed severe, therapy-refractory supraventricular tachycardia and premature beats. In human genetic testing, Gorlin–Goltz syndrome was diagnosed based on a *missense* mutation in the *PTCH1* gene.

Our case shows that, depending on the location and size of the cardiac tumor, different cardiac arrhythmias may occur which influence the child’s risk profile and accordingly their therapeutic management.

## Case report

### First child

The three-year-old girl was born prematurely at 35 + 4 weeks of gestation without complications. At the age of seven months, she became unconscious while playing. She was resuscitated with observed ventricular fibrillation for 30 min in an external clinic. A single defibrillation followed by intubation and a single intratracheal adrenaline dose was performed, whereupon circulation was restored. The lactate level reached a maximum value of 20 mmol/l and the pH value in the venous blood was initially 6.9. After resuscitation, the patient showed sinus rhythm without further need for catecholamines. The patient was transferred to our clinic with stable circulation. The cardiac markers N-terminal fragment brain natriuretic peptides (six days later: 10,780 ng/l) and troponin T (six days later: 640 pg/ml) were significantly elevated for several days.

**Fig. 1:**
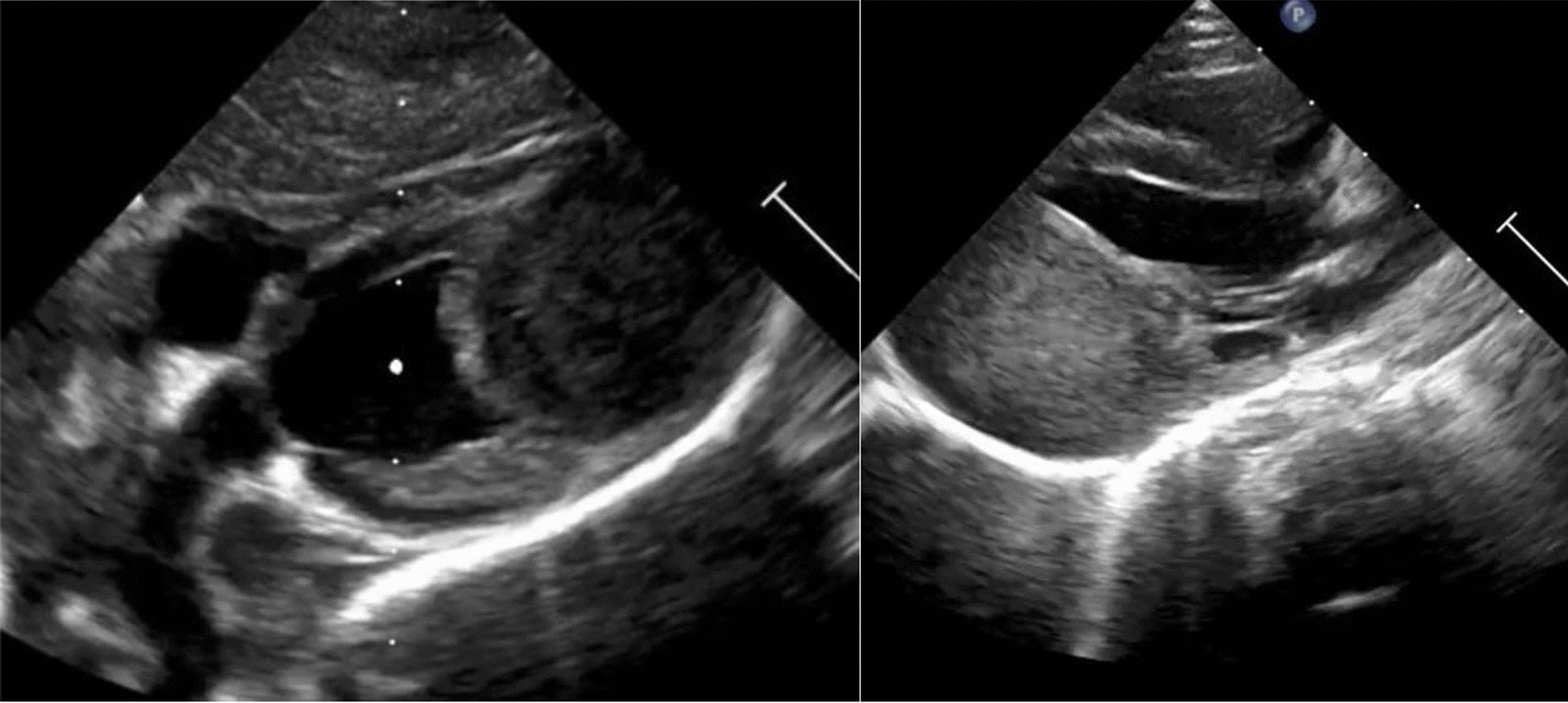
echocardiogram girl. Legend: Two-dimensional echocardiogram showing the tumor arising from the left ventricular wall (modified subcostal view, left) and infiltration of the tumor into the papillary muscle of the mitral valve (parasternal long axis, right)

Subsequent echocardiography demonstrated an apex-near intramural tumor (43 mm × 30 mm × 27 mm) in the posterior and lateral wall of the left ventricle. Ventricular function and contractility were not impaired. There was no obstruction of the left ventricular outflow tract. Diastolic function was moderately reduced. First-degree mitral valve insufficiency due to infiltration of the tumor into the papillary muscle was shown. The coronary arteries were not displaced (See Fig. 1). The cardiac tumor had not been detected prenatally during routine examinations.

**Fig. 2:**
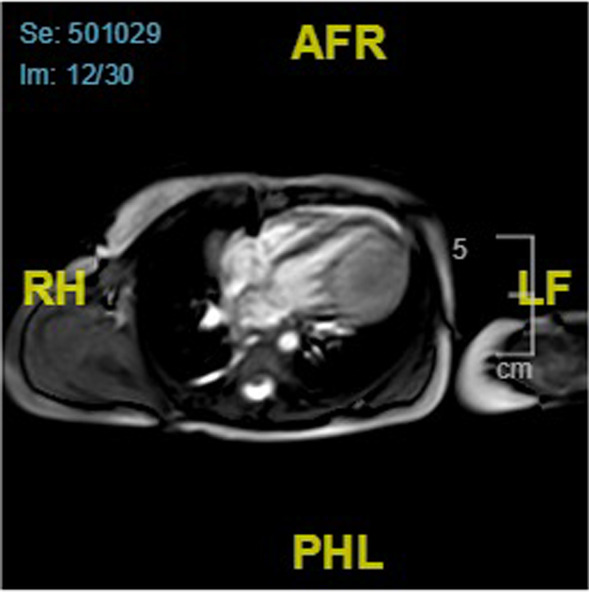
Cardiac magnetic resonance imaging girl. Legend: Cardiac magnetic resonance imaging showing an intracardiac iso- to hypointense mass in the left ventricle in the native T1w-weighted sequence

In cardiac MRI, the mass was hyperintense in the T2-weighted sequence and iso- to hypointense in the native T1w-weighted sequence. It showed minimal late gadolinium enhancement. Calcifications in the tumor were not detected. Visually, the right ventricular function was slightly impaired (See Fig. 2).

The twelve-lead electrocardiography showed negative T-waves in the posterior wall and left precordial leads as a sign of cardiac conduction delay within the tumor mass or unusual repolarization in this area (See Additional files 1). No abnormalities in terms of cardiac arrhythmias, pauses or premature beats were documented in the Holter monitor.

In view of the location and size of the tumor and normal cardiac function without obstruction of the outflow tract, total or partial surgical resection was not performed.

As secondary prevention, a VVI-ICD (Medtronic, model Evera MRI VR SureScan) was implanted by using the “Hsia method” [7]. The lead was placed retrocardial in the transverse pericardial sinus and the ICD generator in the left rectus sheath (See Additional files 2). In addition, the girl takes daily weight-adjusted antiarrhythmic medication at a dose of 2 mg/kg body weight propranolol.

During implantation of the ICD, three biopsies of the tumor were taken, which confirmed the suspected diagnosis of cardiac fibroma. The histology showed cell-rich spindle cell proliferates with isolated calcifications and few blood vessels. There was no cell atypia as an expression of malignancy. In immunohistochemistry, the tumor was positive for α-smooth muscle actin and CD34-antigen. The marker Ki-67 for cell proliferation was not significantly detectable (See Additional files 3).

At the age of 13 months, the girl became unconscious as a result of a hypoglycemic episode (blood glucose 32 mg/dl) during a first attempt at weaning with no nighttime feeding. This event was most likely due to a side effect of the beta-blocker.

Eight months after implantation of the ICD, lead dysfunction of the shock electrode occurred due to a broken cable. The lead was surgically replaced.

In regular echocardiographic examinations, no size progression of the tumor has been detected to date. Regular readings of the implantable cardioverter-defibrillator did not reveal any cardiac arrhythmias leading to delivery of a shock. Otherwise, the girl has developed normally as expected for her age.

### Second child

**Fig. 3:**
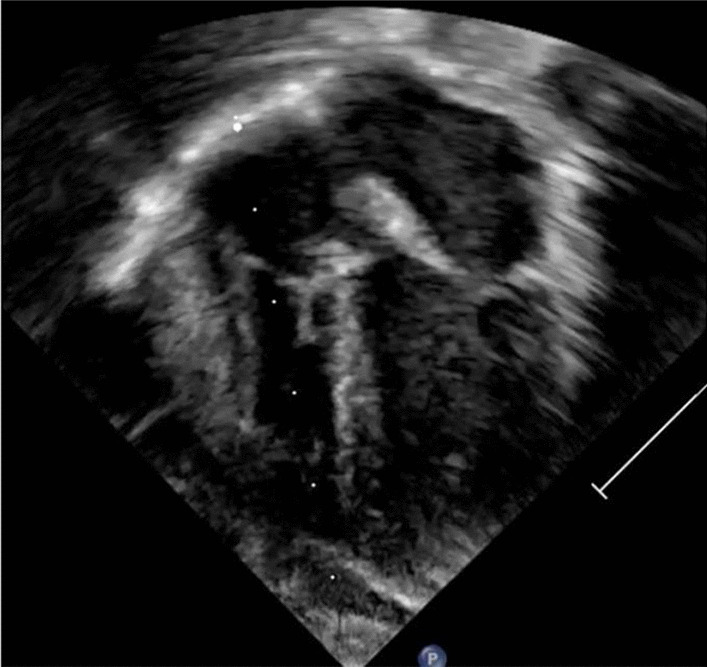
Postnatal two-dimensional echocardiogram. Legend: Four-chamber view showing a mass arising from the right ventricular wall

The seven-month-old brother was born at 38 + 2 weeks of gestation (APAGAR: 9/9/9). Postnatally, an echocardiographic examination was performed in view of the family history. This revealed a mediastinal mass lateral to the right ventricle (2 × 2.5 cm), which had not been visualized prenatally. Apart from the age-related open foramen ovale, there was no obstruction of the right ventricle or limitation of cardiac function (See Fig. 3).

**Fig. 4:**
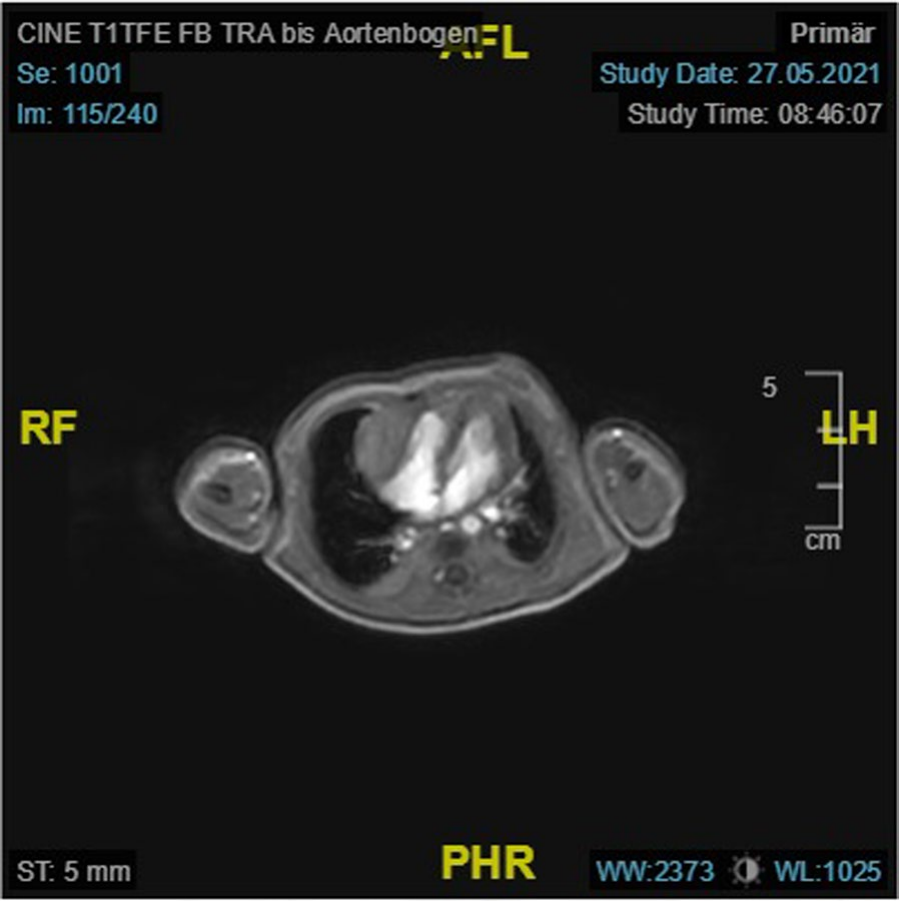
Cardiac magnetic resonance imaging. Legend: Cardiac magnetic resonance imaging showing an isointense mass on the lateral wall of the right ventricle in the T1-weighted sequence

The mediastinal mass was also detected on cardiac MRI. This presented isointense in the T1-weighted sequence and hyperintense in the T2-weighted sequence and showed late gadolinium enhancement, consistent with the characteristics of a cardiac fibroma (See Fig. 4).

In the 24-h electrocardiography, no malignant arrhythmias were detected except for isolated premature beats (< 1% of all beats) (See Additional files 4). The cardiac biomarkers N-terminal fragment brain natriuretic peptides (1896 ng/l) and troponin I (91 pg/ml) were slightly elevated after birth.

Twenty days after birth, the infant was readmitted as an emergency due to a reduction in oxygen saturation (74%) and supraventricular tachycardia (Heart rate: 300/min). Initially, he presented with decreased systemic perfusion with pale skin coloration and prolonged capillary refill time without circulatory instability. No conversion was achieved with multiple doses of adenosine. A subsequent single dose of metoprolol initially resulted in a reduction in heart rate (Heart rate: 150–180/min). Final rate control after further tachycardic episodes was achieved with continuous intravenous therapy with esmolol.

Under subsequent continuous antiarrhythmic therapy with a combination of metoprolol and flecainide, the boy had a strong tendency towards arterial hypotension. In addition, there was an increase in episodes of ventricular arrhythmia and an increasing number of ventricular extrasystoles. These varied between ventricular bigeminy and triplets and supraventricular extrasystoles. Accordingly, the medication was discontinued again and switched to amiodarone. Since then, after a saturation phase with 15 mg/kg body weight, he has received permanent medication with a daily dose of 5 mg/kg body weight amiodarone. Under this therapy, a successful and persistent rhythm control without extrasystoles and cardiac arrhythmias has been achieved so far.

In view of the location and size of the tumor and the normal cardiac function without obstruction of the outflow tract, neither total nor partial surgical resection has been performed.

The children's heart rate and oxygen saturation are monitored at night with a home monitoring system. During daily life, the heart rate of the girl is measured by a smartwatch. The data is transmitted to the parents' smartphone. In addition, the family has an automated external defibrillator at home.

The mother had a sinus thrombosis two years before the birth of the first child with subsequent unremarkable thrombophilia screening. The father had multiple maxillary cysts and plantar pits in adolescence. There is no known case of Gorlin–Goltz syndrome in either family. Clinically and historically, there are no other manifestations of the syndrome at the present time, either in the father nor in the children. Because of this historical evidence and the children's cardiac tumors, the children were tested for Gorlin–Goltz syndrome. In human genetic testing, Gorlin–Goltz syndrome was diagnosed based on a missense mutation in the *PTCH1* gene.

## Discussion

### Different, severe arrhythmias in cardiac fibromas

The case of the siblings demonstrates the occurrence of arrhythmias in children with cardiac fibromas and that these may vary depending on the location of the tumors. Cardiac fibroma is most frequently located in the left ventricle (57.3%), followed by the right ventricle (27.5%) and the interventricular septum (17%). The latter location is associated with increased mortality (58.6%) compared with the other two [8].

Clinically remarkable arrhythmia, such as ventricular tachycardia, occurs in 64% of cases. Cardiac arrest or ventricular fibrillation occurs in 10% of cases. More benign arrhythmias, such as extrasystoles or supraventricular tachycardia, have been described in 4% of cases [2]. Other symptoms may include dyspnea, cyanosis, heart murmurs, cardiomegaly, and heart failure [1]. Such symptoms were not known to have occurred in the girl before the resuscitation event.

Carreon et al. describe re-entry circuits as a cause of ventricular arrhythmias in cardiac fibromas. The substrate of these circuits are interdigitated segments of normal myocardial tissue and pathological tumor tissue, resulting in a delay in cardiac conduction and thus in ventricular arrhythmias. The architecture is similar to the peri-infarct zone after myocardial infarction [9].

In addition, histopathological remodeling processes cause structural changes in the cardiac fibroma with increasing age of the patient. While the degenerate myocardium is the predominant component within the tumor until three years of age, the fibrous component increases more strongly thereafter. A high proportion of degenerated myocardium is associated with a higher risk of cardiac arrest. Therefore, this is a possible reason why children under three years of age with a cardiac fibroma are most likely to suffer a cardiac arrest [9].

The girl was found to have a benign tumor in the apex of the left ventricle after surviving resuscitation. The tumor of the brother overlies the right ventricle from mediastinal and resulted in severe polymorphic supraventricular tachycardia and ventricular premature beats. Dominguez et al. describe a case of sudden infant death syndrome in an eight-month-old boy who became cyanotic and unconscious shortly after feeding. A subsequent forensic examination revealed a cardiac fibroma in the left ventricle [10]. These cases highlight that the earliest possible diagnosis of cardiac fibroma is essential to prevent ventricular tachycardia or sudden infant death syndrome due to cardiac tumor.

Cardiac fibromas are associated with severe arrhythmias, which may vary depending on the location and size of the tumor. Re-entry circuits in the area of the tumor are a possible cause, leading to a disturbance of cardiac conduction. In children with recently developed arrhythmias without a known cause, a cardiac tumor should always be considered.

### Drug therapy with optional ICD implantation in asymptomatic patients

Cardiac fibromas can cause conduction disturbance producing the substrate for life-threatening arrhythmias. In addition, hemodynamically relevant obstruction of the inflow and outflow tract of the heart or obstruction of the coronary arteries may occur. In these cases, depending on the location and size of the tumor and cardiac function, subtotal or total resection of the tumor is indicated. For larger tumors, subtotal resection is preferred over total resection because of the lower risk for intraoperative arrhythmias. Alternatively, heart transplantation can be performed in the case of inoperable findings [1]. Several studies report successful surgery with a very low risk for later ventricular arrhythmias and recurrence of tumor growth [11, 12].

In asymptomatic patients with a cardiac fibroma, the studies are ambiguous regarding therapy. Some authors recommend resection of the tumor because of the risk of ventricular arrhythmia with sudden cardiac death [13]. A few other authors describe drug therapy for asymptomatic patients with antiarrhythmic drugs and, if necessary, ICD implantation as primary or secondary prophylaxis, as an alternative to surgical resection, especially in infants and young children (See Table 1). Complications of ICD in children include lead dysfunction and dislocation, fracture of the shock coil, inducing an inappropriate shock and device infection [14].

**Table 1:** Cases of cardiac fibromas with conservative therapy

Case	Age at diagnosis (gender)	Tumour location	Symptoms	Therapy	Follow-up	References
Case 1	2 weeks (female)	Left ventricle	Asymptomatic, soft systolic murmur, between 2 und 18 months ventricular arrhythmias	Antiarrhythmic medications	After 5 years' follow-up discontinuation of medication, no tumour growth, patient asymptomatic	[15]
Case 2	15 days (female)	Left ventricular apex	Asymptomatic, soft ventricular heart murmur, ECG: ventricular premature beats, inverted T-waves in anterolateral leads	Beta-blocker since the age of 3	6-year follow-up unremarkable	[16]
Case 3	6 months (male)	Right ventricle	Asymptomatic, soft systolic murmur, episode of ventricular tachycardia	Oral therapy with amiodarone	Conservative management for 2 years, afterwards surgical resection because of tumour growth	[11]
Case 4	18 months (male)	Left ventricle	Episode of ventricular tachycardia, otherwise no hemodynamic restrictions	Oral therapy with amiodarone	1-year follow-up unremarkable	[17]
Case 5	6 years (female)	Left ventricle	Episode of ventricular tachycardia	Oral therapy with amiodarone and nadalol	association with Gorlin–Goltz Syndrome, no recurrence of VT at 8 years' follow-up, no tumour growth	[18]

Ünsal and Ekici report the case of a nine-year-old boy who was diagnosed with cardiac fibroma in the wall of the left ventricle in infancy. The tumor was not removed. No tumor growth was detected in regular follow-ups and the boy was otherwise cardiac asymptomatic [16].

Horovitz et al. describe the case of a girl who presented with a systolic murmur at six months of age. After a fibroma in the right ventricle was diagnosed, conservative therapy with amiodarone was initially given. At three years of age, the tumor was surgically removed due to increase in size [11].

In several cases, amiodarone is used to control ventricular arrhythmias. The class III antiarrhythmic drug has a lower rate of side effects in children than in adults. This is due to the more rapid metabolism of amiodarone in children [19]. While side effects occur in 4% of children under ten years of age, those over ten years of age are affected in up to 44% [20]. Side effects include keratopathies, thyroid dysfunction, neurotoxicity, skin changes such as discoloration or photosensitivity, gastrointestinal disturbances, hepatitis, and pulmonary toxicity. In addition, amiodarone may cause worsening of arrhythmia, hypotension, or bradycardic arrhythmia with prolongation of QTc-time. The side effects develop primarily in long-term use in children, whereas short-term use is relatively safe [21].

With combination of flecainide and metoprolol the boy did not suffer any recurrence of supraventricular tachycardia, but nevertheless developed an increasingly higher burden of premature ventricular beats and more importantly up to triplets in the 24-h-holter-electrocardiography. A change of therapy to amiodarone led to stabilization of the arrhythmias. No side effects occurred during the saturation phase of amiodarone and up to the present time.

Since the resuscitation event, the sister has been receiving a daily dose of propranolol. In addition, an ICD was implanted for secondary prevention because of the ventricular fibrillation that had occurred. Except for the hypoglycemic episode and the broken cable of the shock electrode of the defibrillator eight months after implantation, no further complications have occurred to date. Regular follow-up examinations with readings of the ICD revealed no triggering of shocks and or impairment of cardiac function. The cardiac fibroma has not grown so far. There is no obstruction of the inflow and outflow tract. No further cardiac arrhythmias occurred under a daily medical therapy. The girl is otherwise age-appropriately developed and attends kindergarten.

The cases shows that the cardiac arrhythmias can be controlled with individually adjusted drug therapy allows a largely normal life for asymptomatic children with cardiac fibromas. Regular follow-up examinations of the tumor and cardiac function by echocardiography and reading of the ICD are essential.

### Gorlin–Goltz syndrome and cardiac fibromas

In rare cases, cardiac fibromas are associated with Gorlin–Goltz syndrome, an autosomal dominant genetic disease with a variant in the tumor suppressor gene *PTCH1*. The loss-of-function mutation leads to dysfunction of the Sonic Hedgehog signaling pathway in the affected allele on chromosome 9, resulting in excessive activation of a transmembrane protein that influences embryonic development of skin and teeth, tumorigenesis, and organogenesis [22, 23]. More rarely, a variant of the suppressor of fused (*SUFU)* gene is known. Affected individuals with this variant have a significantly higher risk of medulloblastoma in early childhood of 33% compared to affected individuals with the *PTCH1* variant with a risk less than 2% [24].

The syndrome, also known as nevoid basal cell carcinoma syndrome, is a very rare disease with a prevalence of 1:150 000 [25]. An affected parent is present in 70–80% of affected individuals. The other 20–30% have a de novo variant. Affected individuals with the *PTCH1* variant show complete penetrance. Penetrance of the *SUFU* variant is not clearly understood, but reduced penetrance is assumed [24].

The disease is associated with the frequent occurrence of basal cell carcinomas and jaw cysts in 90% of cases before the age of 40 [26]. The most common cutaneous, skeletal, and neurologic changes and associated tumor disease are used as major and minor criteria for clinical diagnosis (See Table 2) [24].

**Table 2:** Major and minor criteria for the diagnosis of GGS [24]

Major criteria	Minor criteria
Lamellar calcification of the falx < age 20 years	Childhood medulloblastoma
Jaw keratocyst	Lympho-mesenteric or pleural cysts
Palmar/plantar pits (≥ 2)	Macrocephaly (Occipitofrontal Circumference > 97th centile)
Basal cell carcinomas (> 5 in a lifetime or 1 basal cell carcinoma before age of 30)	Cleft lip/palate
First-degree relative with NBCCS	Vertebral/rib anomalies
	Preaxial or postaxial polydactyly
	Ovarian/cardiac fibromas
	Ocular anomalies (e.g., cataract)

The presence of two major criteria and one minor criterion or one major criterion and three minor criteria ensure the diagnosis of Gorlin–Goltz syndrome. In addition to multiple basal cell carcinomas, there is a predisposition to medulloblastomas, meningiomas, cardiac and ovarian fibromas, rhabdomyomas, and lymphomas [24].

Human genetic testing includes sequence analysis of the *PTCH1* and *SUFU* genes, deletion and duplication analysis for both genes, and ribonucleic acid analysis of *PTCH1* [24]. If the diagnosis is confirmed, multidisciplinary care is required. This includes regular dermatologic, neurologic, and dental screenings and radiographs to detect skeletal changes. Regular MRI examinations should be performed up to the age of seven for early detection of medulloblastoma. Ultrasound examinations are necessary for the detection of ovarian or cardiac fibromas [22].

The therapy depends individually on the clinical manifestation of the syndrome. Jaw cysts and basal cell carcinomas are usually surgically removed. For basal cell carcinomas, cryotherapy, laser or phototherapy are alternative options depending on the number and size of the tumors. Life expectancy of patients with Gorlin–Goltz syndrome is not significantly reduced [24].

Three to five percent of affected individuals have a cardiac fibroma [6]. A few cases of intracardiac tumors associated with Gorlin–Goltz syndrome have been described in the literature. Ritter et al. report the case of a girl who was found to have a cardiac fibroma in the left ventricle after a first episode of ventricular tachycardia when she was six years old. In addition, she had jaw cysts several times during her childhood and other typical symptoms of Gorlin–Goltz syndrome. At the age of 13 years a variant in the *PTCH1* gene was detected. Her father had a known history of jaw cysts [18].

In our case both siblings have a cardiac tumor. In addition, the father has a history of jaw cysts and plantar pits. For this reason, the girl was tested for Gorlin–Goltz syndrome. In human genetic testing, Gorlin–Goltz syndrome was diagnosed. A variant in the *PTCH1* gene was detected. This case intends to sensitize pediatricians for the diagnosis of a cardiac tumor presenting with different cardiac arrhythmias in association with Gorlin–Goltz syndrome.

### Automated external defibrillators in resuscitation of infants and young children

A Swedish study reported an incidence for out-of-hospital cardiac arrest for children aged zero to 21 years of 4.9 per 100 000 person-years. The survival rate for this group has increased over the past two decades, but is relatively low at 9.3% across all age groups between zero and 21 years. Children under one year of age have the lowest survival rate with 5.1% [27]. The reason for resuscitation in children is more often a respiratory event or shock than a primary cardiac event [28].

In non-professional resuscitation of infants and young children, an automated external defibrillator with pediatric shocks should be used, if available. If such an automated external defibrillator is not available in an emergency, children of all ages should be defibrillated with a standard adult automated external defibrillator [29]. Defibrillators with biphasic shocks are preferred over those with monophasic shocks in children because of the lower defibrillation energy required for terminating ventricular fibrillation or pulseless tachycardia. The recommended dose of a shock in infants and young children is 2–4 J/kg body weight. For an adult automated external defibrillator, the defibrillation energy ranges from 120 to 360 J per shock, which is much higher than the recommended energy for infants and young children [28].

Cardiac tumors are a potential trigger for ventricular arrhythmias or sudden cardiac arrest. Therefore, the family has an automated external defibrillator with pediatric mode. The parents are familiar with pediatric resuscitation. No cardiac emergency requiring defibrillation with the automated external defibrillator has occurred yet.

Bar-Cohen et al. report the first known case of successful resuscitation of a child using a pediatric automated external defibrillator. A four-month-old girl with known Wolff–Parkinson–White syndrome was resuscitated by her parents. The automated external defibrillator induced a biphasic shock of 50 J under ventricular fibrillation. After conversion to asystole and severe bradycardia, normal rhythm was restored within 60 s [30].

European and U.S. guidelines for resuscitation of infants and children up to eight years of age recommend defibrillation with a pediatric automated external defibrillator when manual defibrillation by trained personnel is not available in an emergency [28, 29]. Use of an automated external defibrillator without pediatric attenuators is acceptable (Class IIb recommendation) according to the U.S. guidelines. The benefit is greater than the risk, but the effectiveness is unclear. The recommendation is not based on any scientific studies, but is based on clinical experience of experts [28].

Randomized-controlled trials comparing the benefits of pediatric and adult automated external defibrillators in resuscitation of infants and children up to eight years of age in terms of outcome and neurologic and cardiac consequences for the children are needed to provide evidence-based recommendations for pediatric emergency care in the future.

## Conclusions

The siblings' case report shows that cardiac tumors can be associated with severe arrhythmias. These can vary depending on the location and size of the tumor. In this context, early diagnosis of tumors is essential to prevent serious cardiac events and to initiate preventive therapy.

The benefit of surgical removal of the tumor or long-term conservative therapy depends individually on the location and size of the tumor and impairment of cardiac function. In our case, the girl is well controlled by medication with propranolol and secondary prophylactic ICD implantation. The boy has no ventricular arrhythmias on a daily dose of amiodarone and is cardiac stable. In human genetic testing, Gorlin–Goltz syndrome was diagnosed.

When cardiac fibroma is diagnosed, an association with genetic syndromes, such as Gorlin–Goltz syndrome, should always be considered. Gorlin–Goltz syndrome is a rare disease, but affects patients' risk profile and treatment management.

Children with cardiac tumors have a higher risk of requiring resuscitation due to malignant arrhythmias. In this case, defibrillation with an automated external defibrillator can be a life-saving measure. However, guidelines for the use of automated external defibrillators in resuscitation of infants and young children are not evidence-based and randomized-controlled trials are necessary.

## Supplementary Information

**Additional file 1.** 12 lead Electrocardiogramm (Case 1): QRS axis normal; sinus rhythm; HF: 130/min, PR-Intervall: 150ms, QRS-Time: 70 ms, QT-Time: 300 ms, QTc (Bazett-Formula): 442 ms; cardiac conduction disorders: negative T-waves in II, III, avF, V4-V6.

**Additional file 2.** Thoracic and Abdomen Radiography (Case 1): VVI-ICD generator in the left rectus sheath with retrocardiac placing of the lead in the transverse pericardial sinus “Hsia method” [7].

**Additional file 3.** The histology (Case 1) was provided by the department of pathology, University Medical-Center Hamburg-Eppendorf.

**Additional file 4.** 24-hour holter electrocardiogramm (Case 2) 46 days after birth with detection of several premature beats (2x bigeminus, 1x triplet, 1x couplet).

## Data Availability

Not applicable.

## Abbreviations

ICD: Implantable cardioverter-defibrillator
*PTCH1*: Protein patched homolog 1
*SUFU*: Suppressor of fused
