# Supplementary material for: Siblings with Gorlin–Goltz syndrome associated with cardiac tumors: a case report and review of literature
Source: Orphanet J Rare Dis. 2023 Jul 5;18:178. doi: 10.1186/s13023-023-02792-5 (PMC10324108; doi:10.1186/s13023-023-02792-5)

Supplementary Material 1

12 lead Electrocardiogramm (Case 1): QRS axis normal; sinus rhythm; HF: 130/min, PR-Intervall: 150ms, QRS-Time: 70 ms, QT-Time: 300 ms, QTc (Bazett-Formula): 442 ms; cardiac conduction disorders: negative T-waves in II, III, avF, V4-V6

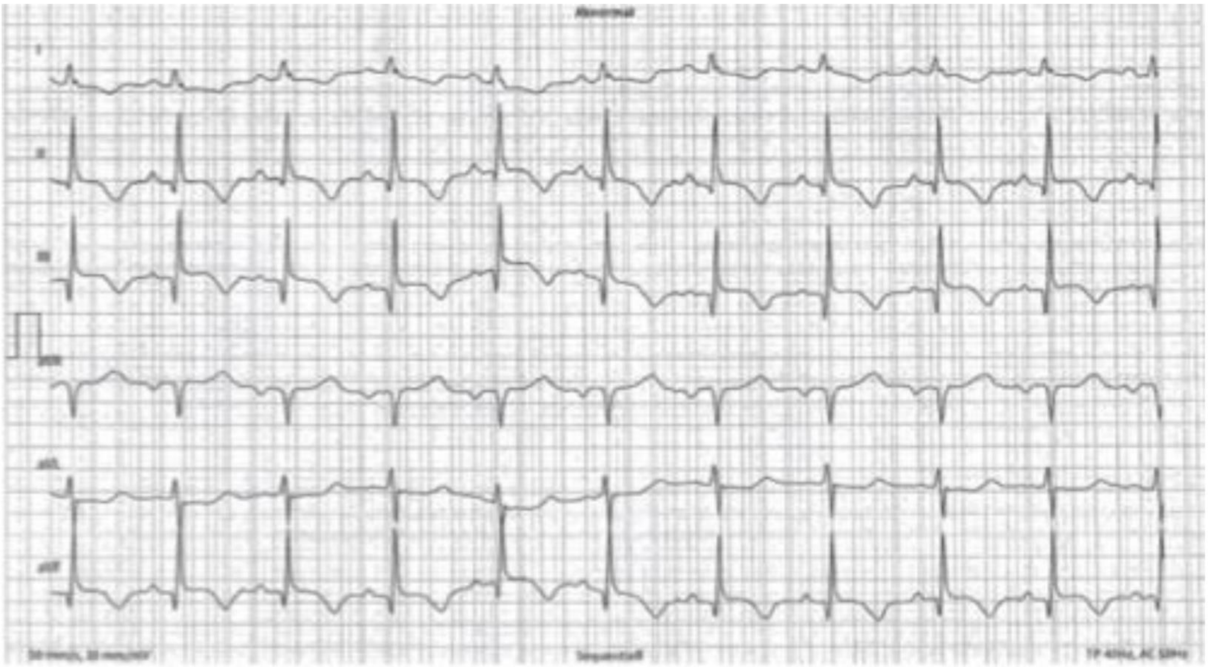

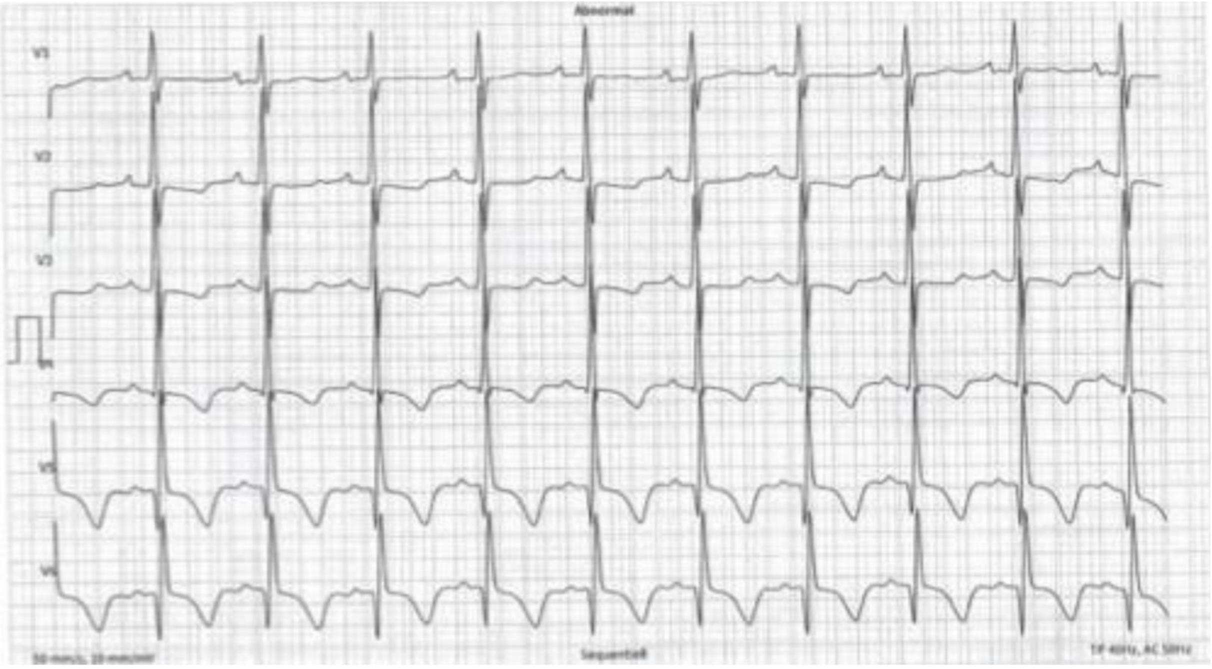

Supplement: Supplementary file 1 — Additional file 1. 12 lead Electrocardiogramm (Case 1): QRS axis normal; sinus rhythm; HF: 130/min, PR-Intervall: 150ms, QRS-Time: 70 ms, QT-Time: 300 ms, QTc (Bazett-Formula): 442 ms; cardiac conduction disorders: negative T-waves in II, III, avF, V4-V6. [file 13023_2023_2792_MOESM1_ESM.pdf]
