# Supplementary material for: Siblings with Gorlin–Goltz syndrome associated with cardiac tumors: a case report and review of literature
Source: Orphanet J Rare Dis. 2023 Jul 5;18:178. doi: 10.1186/s13023-023-02792-5 (PMC10324108; doi:10.1186/s13023-023-02792-5)

Supplementary Material 2

Thoracic and Abdomen Radiography (Case 1): VVI-ICD generator in the left rectus sheath with retrocardiac placing of the lead in the transverse pericardial sinus “Hsia method” [28].

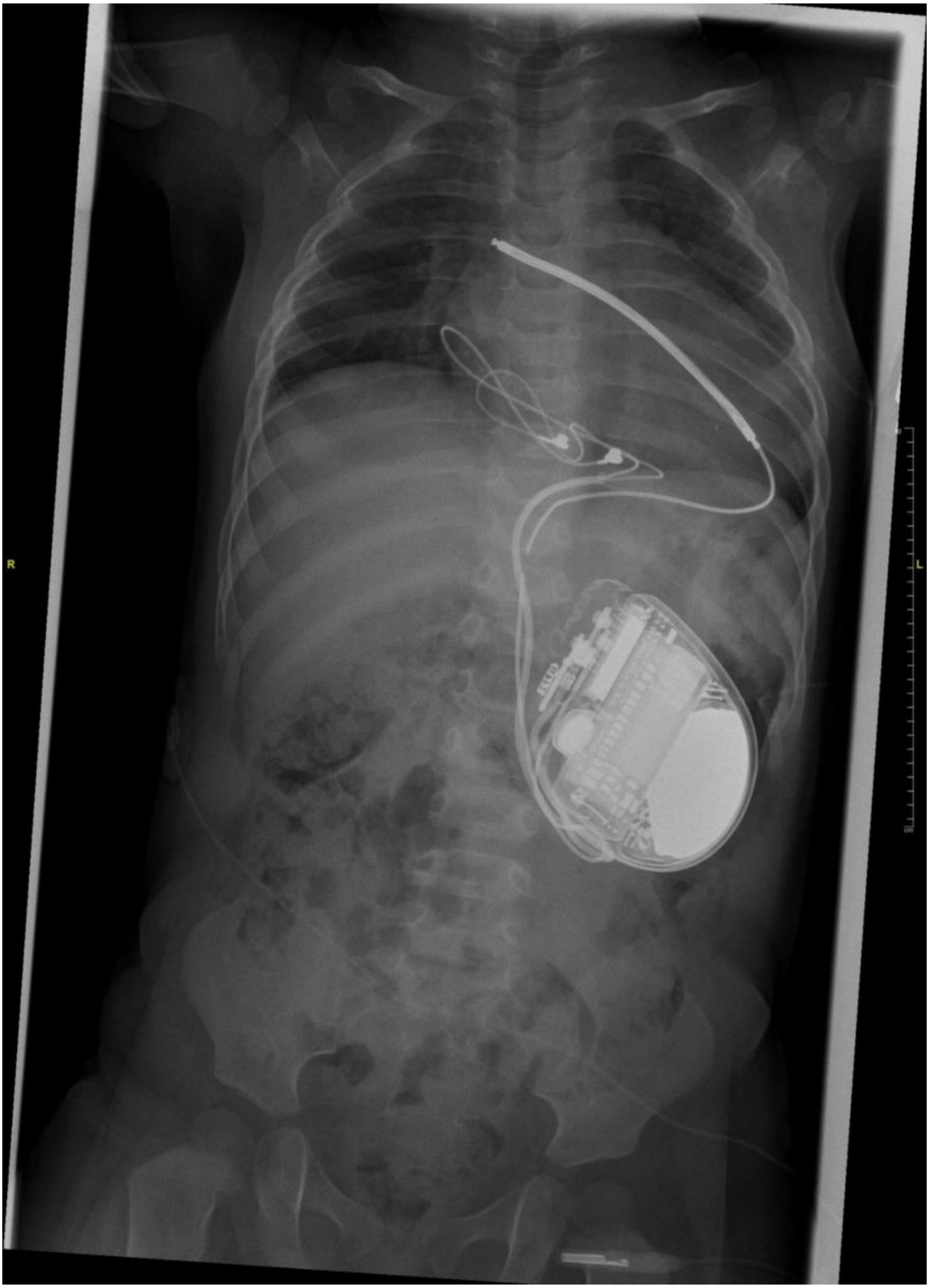

Supplement: Supplementary file 2 — Additional file 2. Thoracic and Abdomen Radiography (Case 1): VVI-ICD generator in the left rectus sheath with retrocardiac placing of the lead in the transverse pericardial sinus “Hsia method” [7]. [file 13023_2023_2792_MOESM2_ESM.pdf]
