# Supplementary material for: Siblings with Gorlin–Goltz syndrome associated with cardiac tumors: a case report and review of literature
Source: Orphanet J Rare Dis. 2023 Jul 5;18:178. doi: 10.1186/s13023-023-02792-5 (PMC10324108; doi:10.1186/s13023-023-02792-5)

Supplementary Material 3

The histology (Case 1) was provided by the department of pathology, University Medical-Center Hamburg-Eppendorf.

Hematoxylin-eosin staining

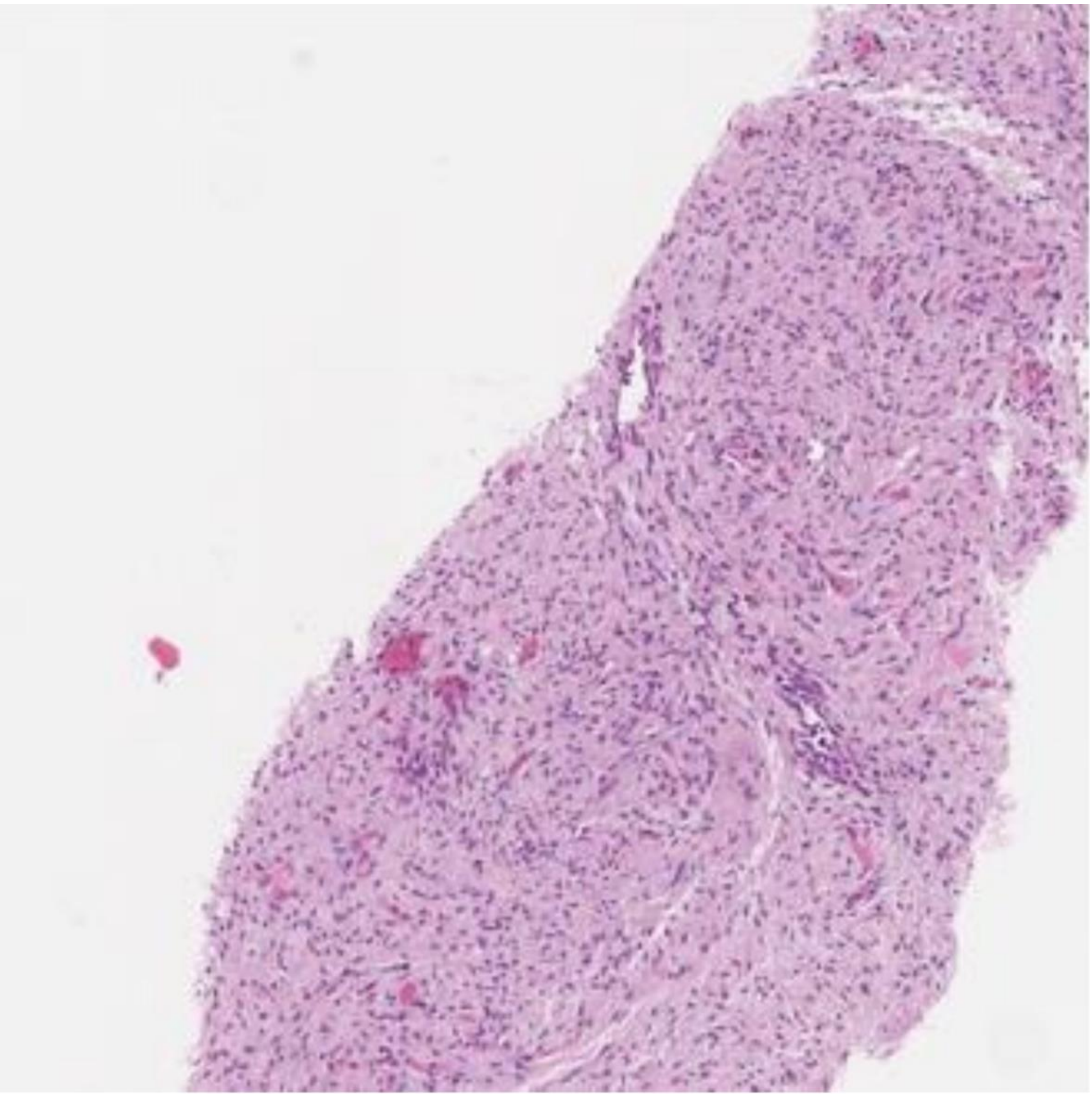

Smooth muscle actin immunhistochemistry

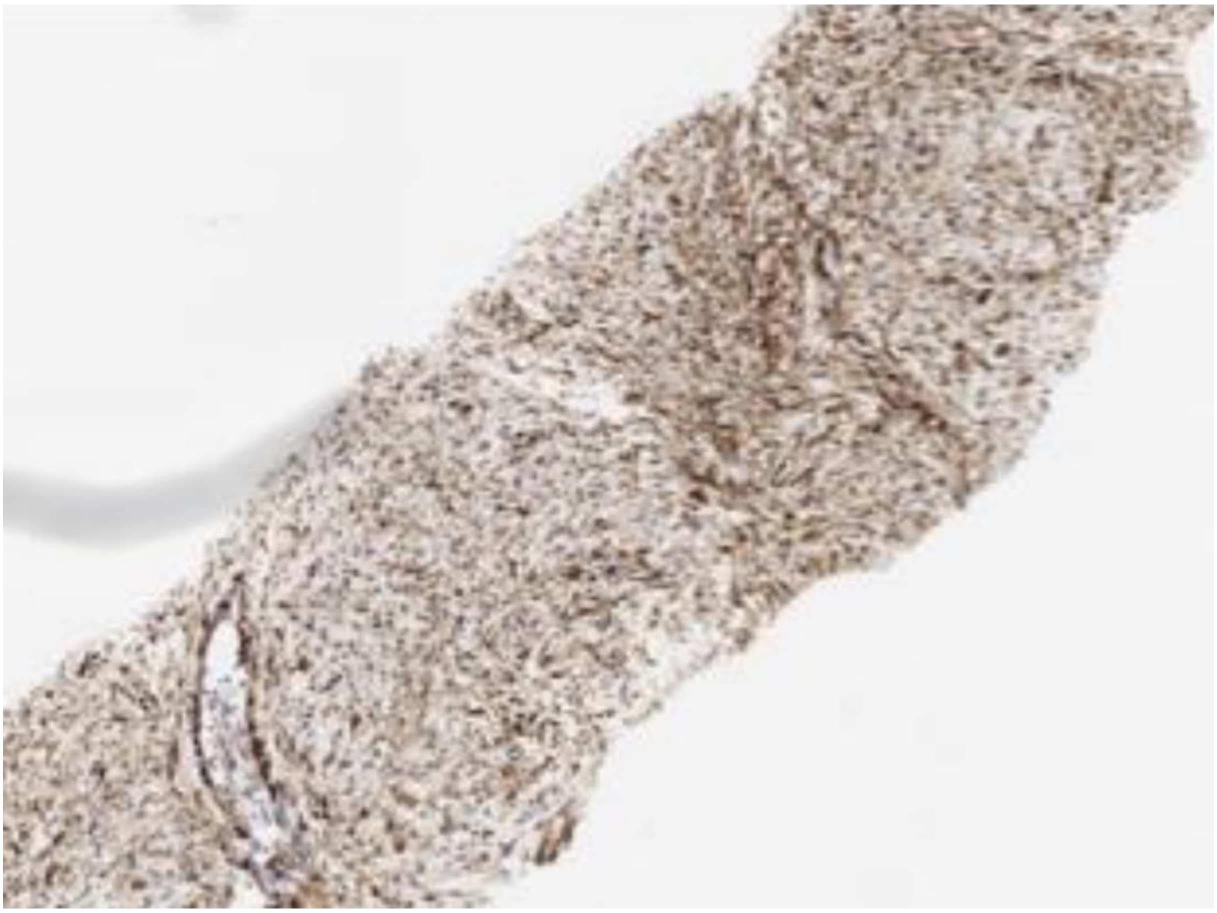

Proliferation marker Ki67 protein

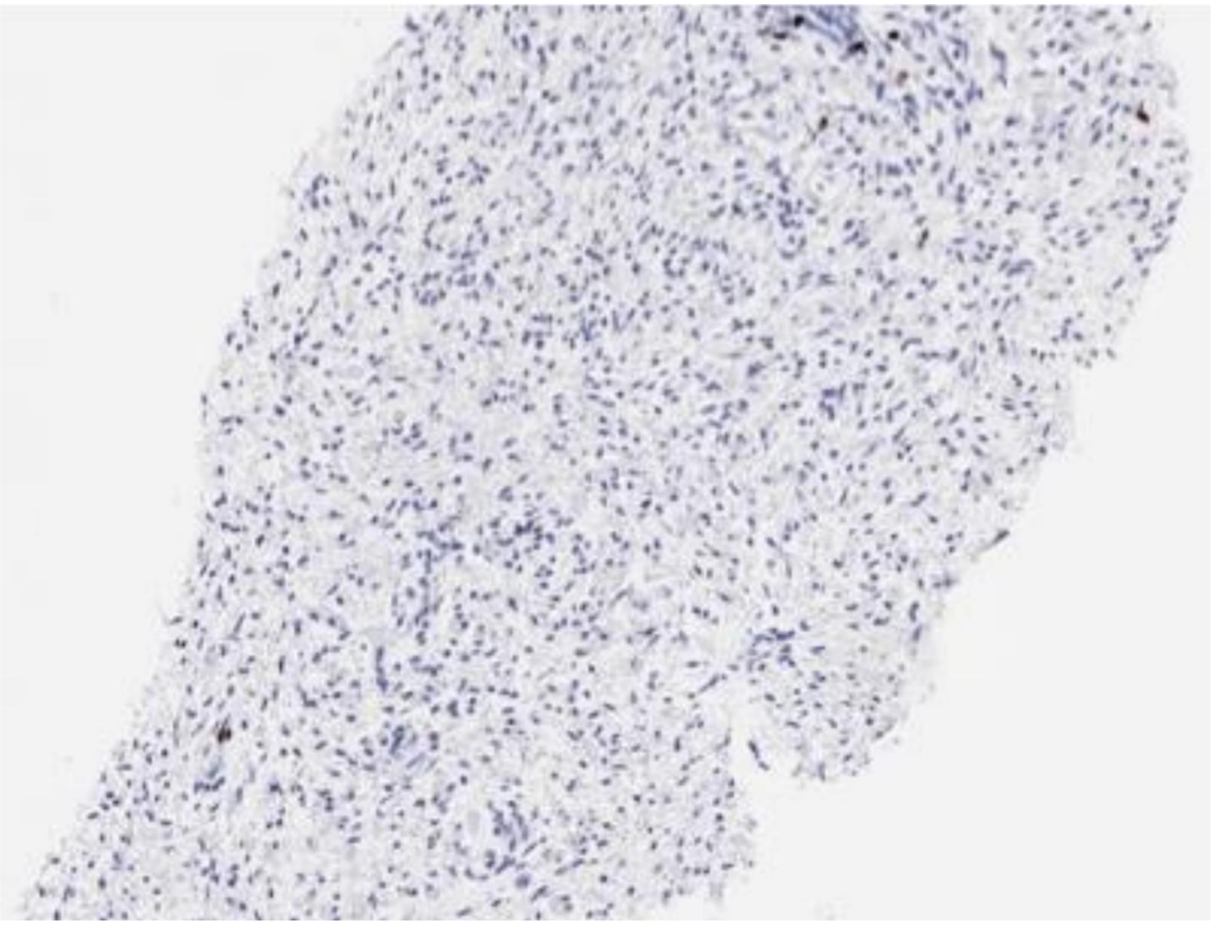

Immunhistochemistry-CD34

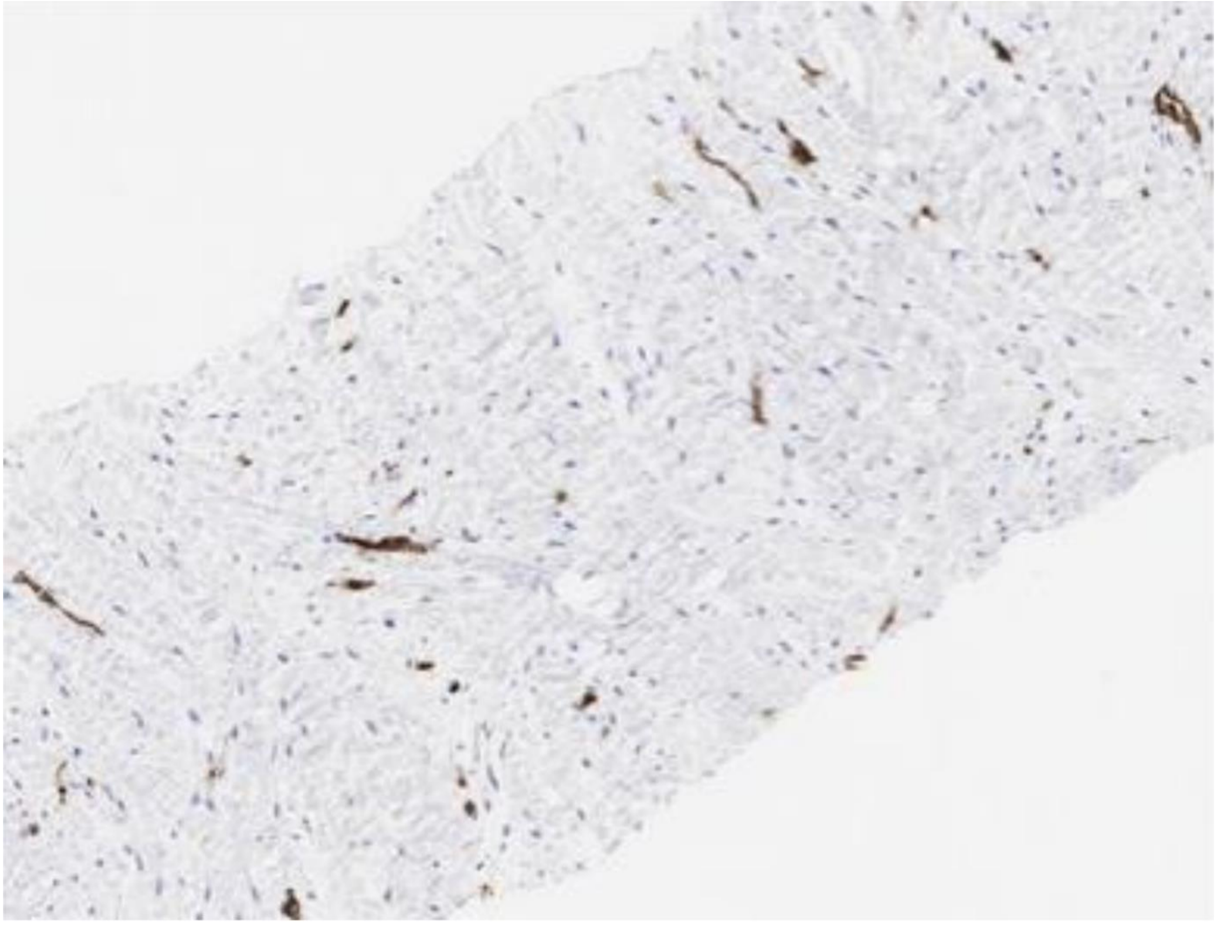

Supplement: Supplementary file 3 — Additional file 3. The histology (Case 1) was provided by the department of pathology, University Medical-Center Hamburg-Eppendorf. [file 13023_2023_2792_MOESM3_ESM.pdf]
