# Supplementary material for: Siblings with Gorlin–Goltz syndrome associated with cardiac tumors: a case report and review of literature
Source: Orphanet J Rare Dis. 2023 Jul 5;18:178. doi: 10.1186/s13023-023-02792-5 (PMC10324108; doi:10.1186/s13023-023-02792-5)

Supplementary Material 4

24-hour holter electrocardiogramm (Case 2) 46 days after birth with detection of several premature beats (2x bigeminus, 1x triplet, 1x couplet).

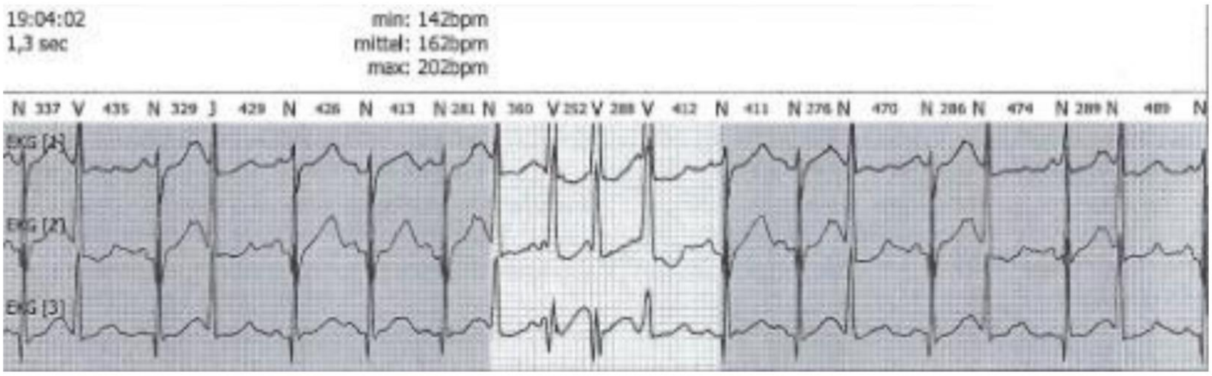

Supplement: Supplementary file 4 — Additional file 4. 24-hour holter electrocardiogramm (Case 2) 46 days after birth with detection of several premature beats (2x bigeminus, 1x triplet, 1x couplet). [file 13023_2023_2792_MOESM4_ESM.pdf]
